# A Logistic Regression Model for Biomechanical Risk Classification in Lifting Tasks

**DOI:** 10.3390/diagnostics12112624

**Published:** 2022-10-29

**Authors:** Leandro Donisi, Giuseppe Cesarelli, Edda Capodaglio, Monica Panigazzi, Giovanni D’Addio, Mario Cesarelli, Francesco Amato

**Affiliations:** 1 Department of Chemical, Materials and Production Engineering, University of Naples Federico II, 80125 Naples, Italy; 2 Institute of Care and Scientific Research Maugeri, 27100 Pavia, Italy; 3 Department of information Technology and Electrical Engineering, University of Naples Federico II, 80125 Naples, Italy

**Keywords:** biomechanical risk assessment, feature extraction, health monitoring, inertial measurement unit, lifting, occupational ergonomics, Revised NIOSH Lifting Equation, statistical learning, wearable sensors, work-related musculoskeletal disorders

## Abstract

Lifting is one of the most potentially harmful activities for work-related musculoskeletal disorders (WMSDs), due to exposure to biomechanical risk. Risk assessment for work activities that involve lifting loads can be performed through the NIOSH (National Institute of Occupational Safety and Health) method, and specifically the Revised NIOSH Lifting Equation (RNLE). Aim of this work is to explore the feasibility of a logistic regression model fed with time and frequency domains features extracted from signals acquired through one inertial measurement unit (IMU) to classify risk classes associated with lifting activities according to the RNLE. Furthermore, an attempt was made to evaluate which are the most discriminating features relating to the risk classes, and to understand which inertial signals and which axis were the most representative. In a simplified scenario, where only two RNLE variables were altered during lifting tasks performed by 14 healthy adults, inertial signals (linear acceleration and angular velocity) acquired using one IMU placed on the subject’s sternum during repeated rhythmic lifting tasks were automatically segmented to extract several features in the time and frequency domains. The logistic regression model fed with significant features showed good results to discriminate “risk” and “no risk” NIOSH classes with an accuracy, sensitivity and specificity equal to 82.8%, 84.8% and 80.9%, respectively. This preliminary work indicated that a logistic regression model—fed with specific inertial features extracted by signals acquired using a single IMU sensor placed on the sternum—is able to discriminate risk classes according to the RNLE in a simplified context, and therefore could be a valid tool to assess the biomechanical risk in an automatic way also in more complex conditions (e.g., real working scenarios).

## 1. Introduction

Musculoskeletal disorders are injuries affecting muscles, bones, nerves, tendons, ligaments, joints, cartilages, spinal discs [1]. According to the National Institute for Occupational Safety and Health (NIOSH), several epidemiological studies have showed a causal relationship between physical exertion at work and work-related musculoskeletal disorders (WMSD) [2]. Several factors have been correlated with WMSD, such as repetitive motion, extreme force, awkward and sustained postures, prolonged sitting and standing [1]. Biomechanical risk factor during physical work is mainly due to three main factors: intensity, repetition and duration [3].

In addition to the more traditional quantitative and semiquantitative observational methods to assess biomechanical risk to which workers are exposed during their work activities [4,5,6,7], wearable sensors are spreading in the occupational ergonomics field since they offer greater agility, precision and duration of measurement [8]. Wearable technologies, in fact, constitute an emerging approach [9] able to support human activities and improve the quality of life [10]. Moreover, these technologies have the power to increase work efficiency among workers, improving their physical well-being and reducing work-related injuries [11,12]. The success of these technologies in the biomechanical risk assessment is due to their capabilities to measure several physiological, kinematic, kinetics parameters, assess human performance, monitor human movements also in a real manufacturing scenario [13,14,15]. Among the wearable technologies, the ones based on inertial measurement units (IMUs) play an important role in the biomechanical risk assessment [16] and they look very promising for occupational medicine and ergonomic applications [17].

Moreover, machine learning (ML) and statistical learning algorithms are gaining popularity also in the ergonomic field, showing a role in the primary prevention of WMSD [18]. Some authors, in fact, have reviewed ML applications closely related to WMSD prevention, such as artificial intelligence for injury risk assessment and performance prediction in team sports [19], fuzzy decision support systems for musculoskeletal disorder diagnoses [20], analysis of occupational accident [21] and textual injury surveillance analyses [22].

Among the work activities involving biomechanical overload, material handling and lifting are one of the most studied in the scientific literature and their causal association with WSMD is widely debated [23]. With a view of prevention, NIOSH established a methodology for assessing biomechanical risk associated with lifting activities [24].

The question remains whether it is possible to classify biomechanical risk classes, computed by means of the Revised NIOSH Lifting Equation (RNLE), using a logistic regression model fed with time and frequency domains features extracted from inertial signals (linear acceleration and angular velocity) acquired by means of a single IMU sensor placed on the subject’s sternum. To this aim, this paper proposes a first application in this direction, detailing a strategy, of potential direct practical application in the context of biomechanical risk assessment (e.g., monitor workers in a real scenario), combing a single IMU sensor and a statistical learning analysis.

## 2. Materials and Methods

### 2.1. IMU-Based Wearable System: The Mobility Lab System

**Figure 1:**
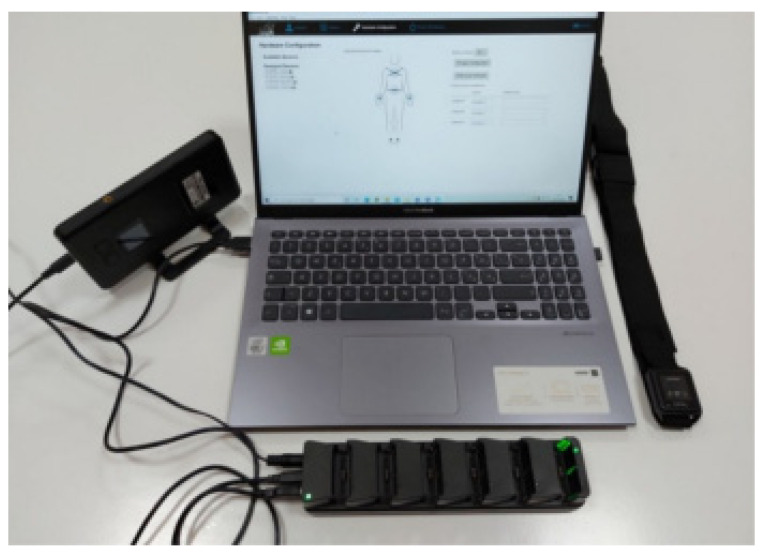
Mobility Lab System: access point, docking station, movement monitor, Mobility Lab software.

**Figure 2:**
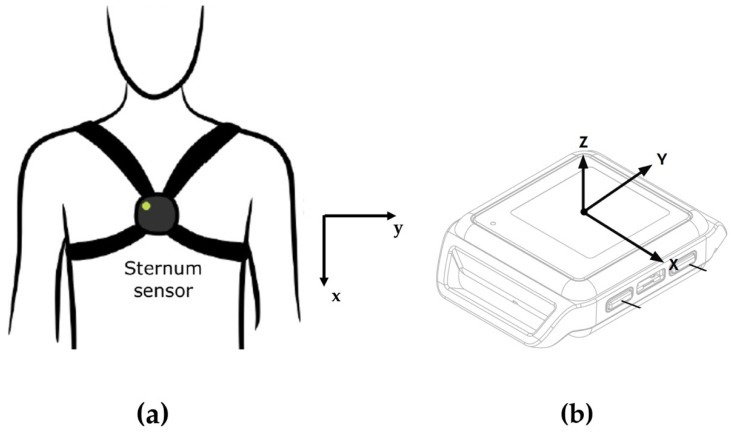
OPAL sensor and sensor positioning sketches. (**a**) OPAL sensor positioning sketch. *Z* axis is orthogonal (positive direction: towards the reader) to the x-y plane. (**b**) Isometric view (sketch) of the external shape of an OPAL sensor. (**a**): reproduced with permission from Martini et al., J. Physiother. Res.; published by Insight Medical Publishing (iMedPub), 2021. (**b**): reproduced with permission from Donisi et al., Sensors; published by Multidisciplinary Publishing Institute (MDPI), 2021.

Mobility Lab System (APDM Inc., Portland, OR, USA) is a commercial IMU-based wearable system for motion capture. The system is composed of both hardware and software. The hardware components are movement monitors, access point, docking station, while the software component consists of a dedicated software named Mobility Lab software (Figure 1). The movement monitors, also named Opal sensors, are essentially IMUs composed of a 3-axes accelerometer with 14-bit resolution, a 3-axes gyroscope with a 16-bit resolution and a 3-axes magnetometer with a 12-bit resolution. The linear acceleration and angular velocity signals are sampled at a frequency of 20 Hz. Opal sensors transfer data by means of a Bluetooth 3.0 communication protocol. The wireless access control point, named access point for short, allows for wireless communication between a host computer and Opal movement monitors. The docking station is used to charge and configure the movement monitors. Finally, the Mobility Lab software is used to configure the hardware components and to record movement data and inertial signals. A single Opal sensor, harnessed with an elastic band on the subject’s sternum (Figure 2), was employed in the present study. The Mobility Lab System has proved to be repeatable [25], reliable [26,27] and accurate [28]. Moreover, its use has appeared in numerous scientific studies [29,30,31,32,33,34,35].

### 2.2. Revised NIOSH Lifting Equation

The RNLE methodology assesses the biomechanical risk to which subjects are exposed during manual lifting of loads [36,37,38]. The following equation—through a multiplicative model with seven variables relating to a lifting task—gives the Recommended Weight Limit (RWL), which is the weight limit for a healthy worker to safely perform a lifting activity:

RWL = LC × HM × VM × DM × AM × FM × GM,(1)

where (see [38] Appendix A1 for a deeper explanation):

- LC: Load Constant 25/20 kg (males, <45/>45 years old, respectively), 20/15 kg (females, <45/>45 years old, respectively);
- HM: Horizontal Multiplier;
- VM: Vertical Multiplier;
- DM: Distance Multiplier;
- AM: Asymmetric Multiplier;
- FM: Frequency Multiplier;
- GM: Grab Multiplier.

By knowing RWL and Actual Weight Lifted (AWL), it is possible to calculate the Lifting Index (LI) as follow:

LI = AWL/RWL,(2)

The LI indicates the potential biomechanical risk associated with lifting activity [39]. In short, LI values less than 1 assume an acceptable situation (absence of potential biomechanical risk) while LI values greater than 1 indicate a potential biomechanical risk, with a risk that increases with increasing LI. In this study, LI values less and greater than 1 were used for the classification analysis.

### 2.3. Study Population

Fourteen healthy volunteers—medical doctors and physiotherapist of the Institute of Care and Scientific Research Maugeri of Montescano (Pavia, Italy)—were enrolled in this study. The participants were not suffering from musculoskeletal disorders or other occupational diseases according to self-reports. Data relating to one participant was not considered in the study, due to the impossibility of segmenting the signal. Table 1 shows the anthropometric characteristics of the study population. The study was approved by the Ethics Committee of the Maugeri Institute. The participants provided written informed consent.

### 2.4. Study Protocol

Each subject performed a task session based on two trials. Each trial consisted of 20 consecutive lifting tasks with a horizontal gripping distance of approximately 40 cm. A pause between the two trials was envisioned to allow subjects to recover before carrying out the next task. Specifically, the first trial consisted of repeated rhythmic lifting of a load in a condition of LI less than 1, named NO RISK class as reported in the Table 2. The second trial consisted of repeated rhythmic lifting of a load in a condition of LI greater than 1, named RISK class as reported in the Table 3. In this preliminary study a simplified scenario was considered, in which the frequency (4/min) and the duration (5 min) of the two lifting trials were constant, while only two variables of the RNLE (load weight and vertical displacement) were manipulated. This was dictated by the intention both to prepare a test that is not excessively demanding for the subjects, and to limit the variability of the factors determining the risk according to the RNLE.

**Figure 3:**
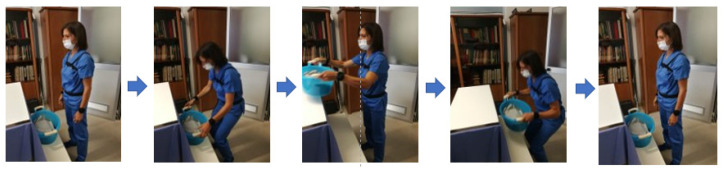
Phases of the lifting tasks: initial rest position, lifting weight with squatting technique upwards, upper destination point, lifting weight with squatting technique downwards, lower destination point and rest position.

A plastic container with weights equally distributed inside was used for the trials. Subjects were instructed to adopt a stable upright posture with the lower limb slightly apart and to perform the squat technique with a two-handed grip. The rhythm of lifting/lowering of the load was indicated acoustically and visually by a digital metronome, signaling the moment of gripping and releasing the load at each act. The phases of the lifting tasks are reported in Figure 3.

### 2.5. Feature Extraction

**Figure 4:**
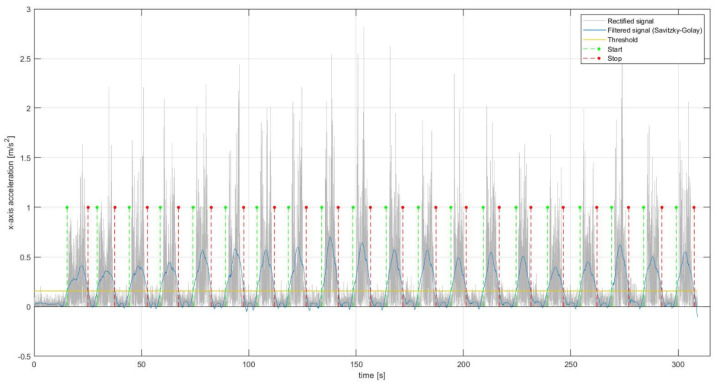
Determination of the ROI (start and end points) corresponding to the lifting by means of the application of Savitzky-Golay filter on the inertial signal (e.g., *x*-axis acceleration) and an empirical threshold.

Inertial signals, namely linear acceleration and angular velocity, underwent a digital signal processing consisting of filtering and segmentation. Inertial signals were filtered with an 8 order Butterworth band-pass filter, with a band pass ranging from 1 Hz to 50 Hz. Successively, the signals were rectified and a Savitzky-Golay filter [40] was applied, choosing a polynomial order equal to 3 and a frame length equal to 1001. Finally, an empirical threshold was set on the signal envelope obtained through the Savitzky-Golay filter to calculate the start and end points (Figure 4) so as to segment the original signal and extract the related Region of Interest (ROI) corresponding to window time during the which the subject performed the lifting.

For each ROI, several features were extracted for each signal (linear acceleration and angular velocity) and for each axis (*x*, *y*, *z*) both in the time and frequency domains. The *x*-axis is the vertical direction, the *y*-axis is the horizontal direction, while the *z*-axis is the direction perpendicular to the sensor plane.

The following time-domain features were extracted:

- Rectified signal area (RSA) [m/s];
- Peak to peak amplitude (PPA) [m/s^2^];
- Mean (MEAN) [m/s^2^];
- Standard Deviation (SD) [m/s^2^];
- Harmonic mean (HM) [m/s^2^];
- 25-percentile (25P) [m/s^2^];
- 75-percentile (75P) [m/s^2^];
- Zero-crossing (ZC) [adim];
- Cumulative length (CL) [m/s^2^];
- Fractal dimension (FD) [adim];
- Number of slope changes (NSC) [adim].

Starting from the power spectrum of the inertial signals computed by means of the fast Fourier transform algorithm, the following frequency-domain features were extracted:

- Entropy (EN) [adim];
- Kurtosis (KU) [adim];
- Skewness (SK) [adim];
- Power (POW) [m/s^2]^;
- Median frequency (MDF) [Hz];
- Mean frequency (MNF) [Hz];
- Peak of the power spectrum (PPS) [m/s];
- Peak frequency (PF) [Hz].

The digital signal processing was performed using MATLAB (MathWorks, R2020a, Natick, MA, USA).

### 2.6. Statistical Learning Analysis

Firstly, a Shapiro-Wilk normality test (Confidence level equal to 95%) was carried out to assess the normality of each feature and consequently to choose a parametric or a non-parametric test. A two-tailed paired *t*-test—if the assumption of normality was assessed—was implemented in order to verify if each feature was differentiated in a significant statistically way between the two classes (RISK, NO RISK) otherwise a non-parametric Wilcoxon test was performed. For both cases, the Confidence level was set to 95% (definition of statistical significance: *p*-value < 0.05).

Secondly, a binary logistic regression was computed to build a predictive model to classify the two risk classes (RISK, NO RISK) according to the RNLE using the features above reported. In order to have a reliable and robust model, the following assumptions were verified [41,42,43]:

1. Absence of multicollinearity;
2. Absence of outliers;
3. Ratio between the sample size of the smallest class and the number of independent variables (the features extracted) greater than 10 [44].

The multicollinearity was solved by means of a correlation study calculating the Pearson correlation coefficient. A feature whose correlation was greater than 0.7 was removed from the binary logistic regression model. The outlier’s detection was performed by computing Cook’s distance and the Center Leverage Value. The features considered in the model creation were the statistically significant ones (*p*-value < 0.05) and the ones with an odd ratio next to 1. The performance of the binary logistic regression model was assessed using the following evaluation metrics: confusion matrix, accuracy, sensitivity and specificity.

Statistical analyses were performed using SPSS Statistics (IBM^®^, v. 28, Armonk, NY, USA).

## 3. Results

First, a statistical analysis by means of a paired *t*-test was performed in order to assess if any differences—between the two biomechanical risk classes (NO RISK, RISK) for each feature—were presented. This analysis was performed for each inertial signal (linear acceleration and angular velocity) acquired by an IMU placed on the sternum and for each axis (*x*, *y*, *z*). Table 4, Table 5 and Table 6 report the results for the linear acceleration along the *x*, *y*, *z* axes, respectively, while Table 7, Table 8 and Table 9 report the results for angular velocity along the *x*, *y*, *z* axes, respectively.

Second, a binary logistic regression model built starting from the features mentioned above was implemented, in order to classify biomechanical risk classes (NO RISK, RISK) according to the RNLE and to study the feasibility of the proposed methods to classify the risk classes. From the correlation analysis, needed to avoid the multicollinearity, 36 out 114 features were used to build the binary logistic regression model considering a threshold of Pearson correlation coefficient equal to 0.7. Moreover, an outlier’s detection was performed by computing Cook’s distance and the Center Leverage Value dimensionless coefficients; 7 instances (3 belonging to the class NO RISK, 4 belonging to the class RISK) out of 520 were removed from the dataset. Successively, we removed from the binary logistic regression model the not statistically significant (*p*-value > 0.05) features and those with an odd ratio close to 1, obtaining a final model fed with 21 out of 36 features. This model respects the condition that the ratio between the sample size (256 instances) of the smallest class (RISK) and the number of independent features (21) is greater than 10 [44]; this ratio resulted in fact greater than 12. Table 10 shows the confusion matrix of the model, while Table 11 reports the evaluation metric scores of the model.

## 4. Discussion

The main objective of the present research paper was to explore the feasibility of a binary logistic regression model—fed with time and frequency domains features extracted from sternum inertial signals (linear acceleration and angular velocity)—to classify biomechanical risk classes associated with lifting activities according to the RNLE.

Moreover, a preliminary statistical analysis based on a paired *t*-test was performed to assess the most discriminative features in classifying risk classes and to understand which inertial signal (linear acceleration, angular velocity) and which axis (*x*, *y*, *z*) were the most representative ones.

The statistical analysis presented in the Table 4, Table 5, Table 6, Table 7, Table 8 and Table 9—based on the paired *t*-test—showed that 96 features out of 114 (84.21%) resulted statistically significant (*p*-value < 0.05) between the two biomechanical risk classes (NO RISK, RISK) underling the discriminative power of the proposed features for the specific objective.

In particular, about the sternum linear acceleration, the statistically significant features were 46 out of 57 (80.70%) while for the sternum angular velocity, the statistically significant features were 50 out of 57 (87.72%). This result would imply that, between the two inertial signals, the angular velocity was more representative compared to linear acceleration to distinguish the two risk classes (NO RISK, RISK) according to the RNLE.

Considering both the linear acceleration and angular velocity, it emerged that for the *x*-axis 35 features out of 38 (92.11%) were statistically significant to discriminate the two risk classes while for the *y*-axis the significant features were 29 out of 38 (76.32%), finally about the *z*-axis the significant features were 32 out of 38 (84.21%). This result would imply that the most representative axis was the *x*-axis, namely the vertical axis along which the lifting movement of the load develops. However, this evidence is not the unique possible one, since in [8] we found the *y* axis (namely, the mediolateral axis) proved informative to discriminate the NIOSH classes, although the load being moved along the *x* axis (namely, the vertical direction).

From this analysis, it also emerged that the HM feature was not statistically significant in distinguishing risk classes for no axis and no inertial signal. On the other hand, the most representative (namely, statistically significant) features, considering both the x, y and z axes and the inertial signals, were PPA, SD, 25P, 75P, MEAN, CL, FD, EN, POW, MDF, and MNF. Though several of these features do not have a clear physical significance, it is known POW and RSA are closely related to energy dissipation during physical activity, while MEAN could provide an idea of how the body is oriented with respect to the direction of gravity. In addition, we report Entropy is used in the activity recognition field; finally, we report also we have proved, in a previous work [8], the importance of SD for similar investigations. 7 out of 11 (63.64%) time-domain features and 4 out of 8 (50%) frequency-domain features emerged among the most representative features. This result would imply that among the selected and extracted features the time-domain features were most representative to discriminate risk classes compared to the frequency-domain ones.

As shown in Table 10, the binary logistic regression model was able to classify correctly 425 out of 513 instances, reaching an overall accuracy of 82.8% (Table 11). This represents a good result and also robust and reliable since the dataset was balanced between the two classes. Moreover, also, the sensitivity and specificity of the regression model reached good results, with a value of 84.8 and 80.9, respectively.

This is the second study that considers risk classification according to the RNLE using a single IMU placed on the subject’s body. In the first study, Donisi et al. [8] used an IMU sensor placed on the lumbar region to acquire linear acceleration and angular velocity on a study population composed of 7 volunteer healthy subjects. In that work [8], the authors extracted only four time-domain features through accurate but time-consuming manual segmentation and implemented sophisticated ML algorithms. The present work, by a simpler model and using an automatic procedure to segment the inertial signals extracting the ROI corresponding to the lifting, reached comparable results in evaluation metrics. Other authors attempted to discriminate biomechanical risk classes according to the RNLE starting from bio-signals. In the work of Varrecchia et al. [45], the authors proposed an artificial neural network—fed with time and frequency domains features extracted from both surface electromyographic signal (sEMG) and signals acquired by means of optoelectronic system—to classify three biomechanical risk classes according to the RNLE reaching an accuracy up to 90%. In another work proposed by the same authors [46], a new feature named Lifting Energy Consumption [47] was used to feed an artificial neural network able to reach an accuracy up to 100%. Even though greater evaluation metric scores were attained in these works [45,46,47] compared to ours, the complex methodologies adopted based on deep learning algorithms, sEMG and optoelectronic systems make this procedure not very applicable in the workplace. Instead, the methodology we propose in this work, based on a single wearable inertial sensor placed on the sternum, is very adapted to monitor the workers’ condition and the potential biomechanical risk to which workers are exposed during work activity (e.g., lifting activity, manual handling).

Mudiyanselage et al. [48] used 2 wireless sEMG muscle sensors placed on thoracic and multifidus muscles to acquire sEMG and therefore to extract some features to feed several ML algorithms, reaching an accuracy greater than 98%. In their work [48], the authors solved the problems of the portability of the system in the workplace while using sEMG—well studied signals in occupational ergonomics but were more prone to noises compared to inertial signals. On the same line of Mudiyanselage et al., Donisi et al. [49] proposed a biomechanical risk classification according to the RNLE using tree-based machine learning algorithms fed with time and frequency domains features extracted from bicep sEMG during lifting activities.

With a similar objective to ours, Brandt et al. [50] attempted to classify lifting activities into low- and high-risk categories according to the guidelines of the Danish Working Environment Authority; using a Linear Discriminant Analysis algorithm, they reached an accuracy of 65%, significantly lower than that achieved by our model.

Results from our study suggest that the automatic segmentation procedure and the combination of time domain and frequency domain features with binary logistic regression model proved to be a valid methodology to assess and monitor the risk of WMSD—according to the RNLE—for manual lifting activities in a relatively low complex context.

## 5. Conclusions

In conclusion, the results showed that the proposed strategy (which combines time and frequency domain features extracted from linear acceleration and angular velocity—acquired by a single IMU placed on the sternum—and a binary logistic regression model) demonstrates early viability—since only two (out of five) RNLE multipliers were modified—to automatically discriminate biomechanical risk classes according to the RNLE during manual handling (e.g., lifting of a load).

This procedure—that includes also an automatic segmentation of the ROI associated to the lifting—is of direct practical relevance for occupational ergonomics, since it presents the opportunity for automatic, economic and non-invasive detection of the risk associated with lifting. Differently from previous literature, which using sEMG and/or optoelectronic methodologies, more suited for a laboratory setting, this strategy has proved potentially applicable in a real working scenario, since the strategy requests only a single IMU sensor.

Future investigation on an enriched dataset that will involve several scenarios and risk classes, also determined by the manipulation of several variables of the RNLE, could confirm the potentiality of the proposed methodology. Moreover, the next step will be to figure out which is the best positioning of the IMU sensor on the human body or, possibly, which is the best combination of positioning of more sensors.

## Figures and Tables

**Table 1:** Anthropometric characteristics of the study population.

Characteristic	
Sex	6 male, 7 female
Age [years]	39.31 ± 12.72
Weight [kg]	67.31 ± 11.35
Height [cm]	171.50 ± 9.13
Manual laterality	10 right, 3 left

Age, Weight and Height are reports as mean ± standard deviation.

**Table 2:** Calculation of LI < 1 as a function of sex, age, load weight, lifting frequency and vertical displacement according to the RNLE.

Subject Sex and Age	Load Weight [kg]	Lifting Frequency [Lifting/min]	Vertical Displacement (Start–End) ^1^ [cm]	LI
Male < 45	6.5	4	70–120	0.57
Male > 45	5.5	4	70–120	0.60
Female	3.5	4	70–120	0.50

^1^ Start and end were calculated considering the handle of the basket and not its base.

**Table 3:** Calculation of LI > 1 as a function of sex, age, load weight, lifting frequency and vertical displacement according to the RNLE.

Subject Sex and Age	Load Weight [kg]	Lifting Frequency [Lifting/min]	Vertical Displacement (Start–End) ^1^ [cm]	LI
Male < 45	12.5	4	20–120	1.48
Male > 45	10.5	4	20–120	1.55
Female	10.5	4	70–120	1.64

^1^ Start and end were calculated considering the handle of the basket and not its base.

**Table 4:** Paired *t*-test NO RISK/RISK classes for each feature extracted from *x*-axis acceleration (ax).

Features *	NO RISK Mean ± Std	RISK Mean ± Std	*p*-Value
RSAax	405.33 ± 110.37	464.06 ± 116.47	<0.001
PPAax	4.15 ± 1.47	4.79 ± 1.26	<0.001
SDax	0.57 ± 0.17	0.64 ± 0.14	<0.001
HMax	0.44 ± 5.47	0.87 ± 19.09	0.1340
75Pax	0.22 ± 0.05	0.26 ± 0.05	<0.001
25Pax	−0.26 ± 0.06	−0.31 ± 0.07	<0.001
MEANax	0.14 ± 0.04	0.17 ± 0.05	<0.001
ZCax	78.71 ± 20.28	84.75 ± 21.76	<0.001
CLax	75.50 ± 24.03	99.72 ± 26.92	<0.001
FDax	1.00 ± 0.00	1.01 ± 0.00	<0.001
NSCax	319.31 45.20	324.22 50.43	0.0076
ENax	0.51 ± 0.06	0.55 ± 0.05	<0.001
KUax	132.80 59.07	118.81 59.99	<0.001
SKax	10.31 ± 2.27	9.58 ± 2.39	<0.001
POWax	186.96 109.31	228.56 116.66	<0.001
MDFax	1.87 ± 0.51	2.20 ± 0.71	<0.001
MNFax	3.01 ± 0.94	3.66 ± 1.10	<0.001
PPSax	21.21 ± 15.03	21.89 ± 12.77	0.149
PFax	1.38 ± 0.27	1.53 0.35	<0.001

Definition of statistical significance: *p*-value < 0.05. * Rectified signal area (RSA); Peak to peak amplitude (PPA); Mean (MEAN); Standard Deviation (SD); Harmonic mean (HM); 25-percentile (25P); 75-percentile (75P); Zero-crossing (ZC); Cumulative length (CL); Fractal dimension (FD); Number of slope changes (NSC); Entropy (EN); Kurtosis (KU); Skewness (SK); Power (POW); Median frequency (MDF); Mean frequency (MNF); Peak of the power spectrum (PPS); Peak frequency (PF).

**Table 5:** Paired *t*-test NO RISK/RISK classes for each feature extracted from *y*-axis acceleration (ay).

Features *	NO RISK Mean ± Std	RISK Mean ± Std	*p*-Value
RSAay	143.70 ± 44.34	182.36 ± 64.92	<0.001
PPAay	1.60 ± 0.57	2.15 ± 0.75	<0.001
SDay	0.20 ± 0.06	0.25 ± 0.08	<0.001
HMay	0.05 ± 0.74	0.91 ± 8.13	0.939
75Pay	0.09 ± 0.02	0.11 ± 0.03	<0.001
25Pay	−0.09 ± 0.03	−0.11 ± 0.03	<0.001
MEANay	0.38 ± 0.10	0.44 ± 0.09	<0.001
ZCay	138.27 ± 24.54	137.88 ± 23.58	0.6520
Clay	50.77 ± 17.97	64.91 ± 23.79	<0.001
FDay	1.00 ± 0.00	1.01 ± 0.00	<0.001
NSCay	412.34 ± 50.82	407.90 ± 56.37	0.9780
ENay	0.65 ± 0.03	0.66 ± 0.03	<0.001
KUay	49.79 ± 31.04	48.03 ± 32.89	0.1230
SKay	5.85 ± 1.68	5.68 ± 1.76	0.0550
POWay	21.96 ± 15.90	36.68 ± 30.18	<0.001
MDFay	5.41 ± 1.96	5.67 ± 2.11	0.0130
MNFay	6.85 ± 1.41	7.04 ± 1.59	<0.001
PPSay	0.91 ± 0.65	1.52 ± 2.26	<0.001
PFay	3.44 ± 2.71	3.62 ± 2.94	0.9600

Definition of statistical significance: *p*-value < 0.05. * Rectified signal area (RSA); Peak to peak amplitude (PPA); Mean (MEAN); Standard Deviation (SD); Harmonic mean (HM); 25-percentile (25P); 75-percentile (75P); Zero-crossing (ZC); Cumulative length (CL); Fractal dimension (FD); Number of slope changes (NSC); Entropy (EN); Kurtosis (KU); Skewness (SK); Power (POW); Median frequency (MDF); Mean frequency (MNF); Peak of the power spectrum (PPS); Peak frequency (PF).

**Table 6:** Paired *t*-test NO RISK/RISK classes for each feature extracted from *z*-axis acceleration (az).

Features *	NO RISK Mean ± Std	RISK Mean ± Std	*p*-Value
RSAaz	260.34 ± 75.91	349.12 ± 89.93	<0.001
PPAaz	3.12 ± 1.21	4.30 ± 1.48	<0.001
SDaz	0.36 ± 0.11	0.49 ± 0.13	<0.001
HMaz	0.08 ± 1.23	0.08 ± 2.34	0.6760
75Paz	0.16 ± 0.04	0.21 ± 0.06	<0.001
25Paz	−0.16 ± 0.04	−0.22 ± 0.06	<0.001
MEANaz	0.25 ± 0.07	0.33 ± 0.08	<0.001
ZCaz	119.74 ± 32.32	123.01 ± 37.84	0.0470
CLaz	79.73 ± 32.82	109.49 ± 39.40	<0.001
FDaz	1.00 ± 0.00	1.01 ± 0.00	<0.001
NSCaz	418.80 ± 51.49	420.51 ± 73.58	0.1560
ENaz	0.59 ± 0.06	0.61 ± 0.06	<0.001
KUaz	143.35 ± 68.52	118.38 ± 60.15	<0.001
SKaz	10.48 ± 2.62	9.39 ± 2.59	<0.001
POWaz	74.16 ± 46.93	134.43 ± 83.27	<0.001
MDFaz	3.21 ± 2.22	3.61 ± 2.67	<0.001
MNFaz	5.21 ± 1.67	5.64 ± 1.91	<0.001
PPSaz	7.01 ± 4.79	11.39 ± 9.24	<0.001
PFaz	1.32 ± 0.24	1.63 ± 1.83	0.6950

Definition of statistical significance: *p*-value < 0.05. * Rectified signal area (RSA); Peak to peak amplitude (PPA); Mean (MEAN); Standard Deviation (SD); Harmonic mean (HM); 25-percentile (25P); 75-percentile (75P); Zero-crossing (ZC); Cumulative length (CL); Fractal dimension (FD); Number of slope changes (NSC); Entropy (EN); Kurtosis (KU); Skewness (SK); Power (POW); Median frequency (MDF); Mean frequency (MNF); Peak of the power spectrum (PPS); Peak frequency (PF).

**Table 7:** Paired *t*-test NO RISK/RISK classes for each feature extracted from *x*-axis angular velocity (vx).

Features *	NO RISK Mean ± Std	RISK Mean ± Std	*p*-Value
RSAvx	44.04 ± 10.68	53.42 ± 13.44	<0.001
PPAvx	0.46 ± 0.19	0.60 ± 0.19	<0.001
SDvx	0.06 ± 0.01	0.07 ± 0.02	<0.001
HMvx	0.48 ± 7.14	0.07 ± 0.50	0.5010
75Pvx	0.02 ± 0.01	0.03 ± 0.01	<0.001
25Pvx	−0.02 ± 0.01	−0.03 ± 0.01	<0.001
MEANvx	0.04 ± 0.01	0.05 ± 0.01	<0.001
ZCvx	82.54 ± 18.53	89.13 ± 21.74	<0.001
CLvx	10.16 ± 3.46	13.30 ± 4.15	<0.001
FDvx	1.00 ± 0.00	1.01 ± 0.00	<0.001
NSCvx	313.75 ± 44.28	321.82 ± 51.81	0.0030
ENvx	0.56 ± 0.05	0.58 ± 0.04	<0.001
KUvx	125.56 ± 62.74	107.40 ± 54.84	<0.001
SKvx	9.79 ± 2.37	8.96 ± 2.22	<0.001
POWvx	1.94 ± 1.06	2.85 ± 1.35	<0.001
MDFvx	2.36 ± 0.58	2.71 ± 0.76	<0.001
MNFvx	3.87 ± 0.86	4.41 ± 1.01	<0.001
PPSvx	0.19 ± 0.11	0.23 ± 0.14	<0.001
PFvx	1.58 ± 0.38	1.67 ± 0.42	0.0010

Definition of statistical significance: *p*-value < 0.05. * Rectified signal area (RSA); Peak to peak amplitude (PPA); Mean (MEAN); Standard Deviation (SD); Harmonic mean (HM); 25-percentile (25P); 75-percentile (75P); Zero-crossing (ZC); Cumulative length (CL); Fractal dimension (FD); Number of slope changes (NSC); Entropy (EN); Kurtosis (KU); Skewness (SK); Power (POW); Median frequency (MDF); Mean frequency (MNF); Peak of the power spectrum (PPS); Peak frequency (PF).

**Table 8:** Paired *t*-test NO RISK/RISK classes for each feature extracted from *y*-axis angular velocity (vy).

Features *	NO RISK Mean ± Std	RISK Mean ± Std	*p*-Value
RSAvy	109.69 ± 28.76	135.25 ± 34.67	<0.001
PPAvy	1.34 ± 0.56	1.59 ± 0.69	<0.001
SDvy	0.15 ± 0.04	0.19 ± 0.05	<0.001
HMvy	0.21 ± 1.88	−0.21 ± 4.16	0.5380
75Pvy	0.07 ± 0.02	0.08 ± 0.02	<0.001
25Pvy	−0.07 ± 0.02	−0.09 ± 0.02	<0.001
MEANvy	0.10 ± 0.03	0.13 ± 0.03	<0.001
ZCvy	68.85 ± 24.58	74.23 ± 30.07	<0.001
CLvy	22.95 ± 11.30	29.77 ± 10.97	<0.001
FDvy	1.00 ± 0.00	1.01 ± 0.00	<0.001
NSCvy	324.49 ± 52.89	334.78 ± 68.65	<0.001
ENvy	0.49 ± 0.07	0.51 ± 0.07	<0.001
KUvy	194.42 ± 74.38	199.58 ± 86.44	0.5540
SKvy	12.65 ± 2.63	12.77 ± 2.99	0.6830
POWvy	13.37 ± 7.19	19.71 ± 11.09	<0.001
MDFvy	1.68 ± 0.52	1.86 ± 0.99	0.0140
MNFvy	3.20 ± 1.17	3.67 ± 1.43	<0.001
PPSvy	1.97 ± 1.11	2.88 ± 1.95	<0.001
PFvy	1.27 ± 0.18	1.24 ± 0.19	0.0460

Definition of statistical significance: *p*-value < 0.05. * Rectified signal area (RSA); Peak to peak amplitude (PPA); Mean (MEAN); Standard Deviation (SD); Harmonic mean (HM); 25-percentile (25P); 75-percentile (75P); Zero-crossing (ZC); Cumulative length (CL); Fractal dimension (FD); Number of slope changes (NSC); Entropy (EN); Kurtosis (KU); Skewness (SK); Power (POW); Median frequency (MDF); Mean frequency (MNF); Peak of the power spectrum (PPS); Peak frequency (PF).

**Table 9:** Paired *t*-test NO RISK/RISK classes for each feature extracted from *z*-axis angular velocity (vz).

Features *	NO RISK Mean ± Std	RISK Mean ± Std	*p*-Value
RSAvz	32.94 ± 9.08	43.54 ± 13.65	<0.001
PPAvz	0.33 ± 0.11	0.49 ± 0.16	<0.001
SDvz	0.04 ± 0.01	0.06 ± 0.02	<0.001
HMvz	−0.01 ± 0.28	−0.01 ± 0.25	0.2170
75Pvz	0.02 ± 0.01	0.03 ± 0.01	<0.001
25Pvz	−0.02 ± 0.01	−0.03 ± 0.01	<0.001
MEANvz	0.03 ± 0.01	0.04 ± 0.01	<0.001
ZCvz	75.30 ± 14.85	79.12 ± 17.35	<0.001
CLvz	7.14 ± 2.29	10.08 ± 3.31	<0.001
FDvz	1.00 ± 0.00	1.01 ± 0.00	<0.001
NSCvz	284.87 ± 40.98	287.71 ± 46.58	0.0030
ENvz	0.56 ± 0.04	0.58 ± 0.04	<0.001
KUvz	99.84 ± 49.40	98.46 ± 51.43	0.4540
SKvz	8.67 ± 2.10	8.50 ± 2.17	0.1970
POWvz	1.11 ± 0.62	2.00 ± 1.23	<0.001
MDFvz	2.66 ± 0.69	2.92 ± 0.79	<0.001
MNFvz	3.83 ± 0.66	4.24 ± 0.90	<0.001
PPSvz	0.09 ± 0.06	0.16 ± 0.12	<0.001
PFvz	1.72 ± 0.66	1.85 ± 0.78	0.0430

Definition of statistical significance: *p*-value < 0.05. * Rectified signal area (RSA); Peak to peak amplitude (PPA); Mean (MEAN); Standard Deviation (SD); Harmonic mean (HM); 25-percentile (25P); 75-percentile (75P); Zero-crossing (ZC); Cumulative length (CL); Fractal dimension (FD); Number of slope changes (NSC); Entropy (EN); Kurtosis (KU); Skewness (SK); Power (POW); Median frequency (MDF); Mean frequency (MNF); Peak of the power spectrum (PPS); Peak frequency (PF).

**Table 10:** Confusion Matrix of the Binary Logistic Regression Model.

	NO RISK	RISK	Percentage of Correctness [%]
**NO RISK**	218	39	84.8
**RISK**	49	207	80.9

**Table 11:** Evaluation metric scores of the Binary Logistic Regression Model expressed in percentage value.

Accuracy [%]	Sensitivity [%]	Specificity [%]
82.8	84.8	80.9

## Data Availability

The datasets generated and analyzed in this study are not publicly available due to privacy policy, but are available from the corresponding author on reasonable request.
